# Surgical Considerations of One-Stage Reconstruction of Large Extremity Defects Using a Thin Deep Inferior Epigastric Perforator Flap

**DOI:** 10.1055/a-1976-2212

**Published:** 2023-02-01

**Authors:** Seung Yeol Lee, Moon Chul Seok, Bo Young Park

**Affiliations:** 1 Department of Orthopedic Surgery, Myongji Hospital, Hanyang University College of Medicine, Gyeonggi, Korea; 2 Department of Plastic and Reconstructive Surgery, School of Medicine, Ewha Womans University, Seoul, Republic of Korea

**Keywords:** perforator flap, large defect reconstruction, deep inferior epigastric perforator flap

## Abstract

**Background** One-stage reconstruction with “thin perforator flaps” has been attempted to salvage limbs and restore function. The deep inferior epigastric perforator (DIEP) flap is a commonly utilized flap in breast reconstruction (BR). The purpose of this study is to present the versatility of DIEP flaps for the reconstruction of large defects of the extremities.

**Methods** Patients with large tissue defects on extremities who were treated with thin DIEP flaps from January 2016 to January 2018 were included. They were minimally followed up for 36 months. We analyzed the etiology and location of the soft tissue defect, flap design, anastomosis type, outcome, and complications. We also considered the technical differences in the DIEP flap between breast and extremity reconstruction.

**Results** Overall, six free DIEP flaps were included in the study. The flap size ranged from 15 × 12 to 30 × 16 cm ^2^ . All flaps were transversely designed similar to a traditional BR design. Three flaps were elevated with two perforators. Primary closure of the donor site was possible in all cases. Five flaps survived with no complications. However, partial necrosis occurred in one flap.

**Conclusion** A DIEP flap is not the first choice for soft tissue defects, but it should be considered for one-stage reconstruction of large defects when the circulation zone of the DIEP flap is considered. In addition, this flap has many advantages over other flaps such as provision of the largest skin paddle, low donor site morbidity with a concealed scar, versatile supercharging technique, and a long pedicle.

## Introduction

Large soft tissue defects of the extremities are difficult to cover, even though various options have been suggested. Perforator flaps have been shown to precisely match the defect and provide good functional and aesthetic outcomes in extremity reconstruction. 1 However, increasing flap size is a risk factor for major complications and flap failures in large defects of the extremities. It has been suggested that a larger flap dimension is associated with higher metabolic demand and is more sensitive to vascular injury; therefore, it is a risk factor for increased complications. In a review of 112 free flaps for open tibial fractures, the complication rate increased threefold when the size of defect was more than 200 cm ^2^ . 2 Hence, perforator flaps have not gained widespread acceptance for large defects of the extremities, even though recent advances have streamlined this method of extremity reconstruction.

The deep inferior epigastric perforator (DIEP) flap is the most commonly utilized flap in breast reconstruction (BR). 3 It provides a single free flap reconstruction for large-sized defects without the need of a second free flap or skin grafting of donor sites, which might result in poor functional and aesthetic outcomes postoperatively. Although advantages were found with a one-stage coverage with DIEP flap in large extremity defects, some technical tips such as flap circulation and its bulkiness compared to other perforator flaps should be considered during the procedure.

In this report, we have elaborated on why the DIEP has not gained popularity in extremity reconstruction. Subsequently, we have suggested technical tips to overcome the disadvantages of DIEP flaps when used in extremity reconstruction compared to that with BR. We have also presented our modified DIEP flap coverage for large extremity defects, which was harvested in the same manner and was thinned intraoperatively after microanastomosis with good functional and aesthetic outcomes.

## Methods

The study was performed in accordance with the tenets of the Declaration of Helsinki and approved by the institutional ethics committee of the Ewha Womans University Seoul Hospital (2021-07-039). This study included patients who presented with large tissue defects (over than 15 cm × 10 cm) on their extremities and were treated with thin DIEP flaps from January 2016 to January 2018 that were followed up for more than 36 months. All of the patients provided written informed consent. A retrospective analysis was performed using operation records and clinical follow-up data. Flap size, number of perforators, anastomosis patterns, complications, and final outcomes were analyzed.

### Surgical Method

The overall surgical method is similar to that of DIEP in BR. The flap was designed transversely to enable primary closure of the donor site. Massive debridement before reconstruction was mandatory. The preoperative planning was the most important process. The dimensions of the flap could be determined before the surgery; likewise, the volume of the DIEP flap in BR was also considered. In BR, when the inset rate (volume) is greater than 75% of the whole flap, harvesting a bipedicle DIEP flap or supercharging is recommended with due consideration to the perfusion zone. 4 The patients underwent preoperative contrast-enhanced computed tomographic angiography (CTA) for perforator identification; this is not routinely performed in BR. The number and location of the perforators were marked with CTA. The perforators were detected and marked with a handheld Doppler. The transverse design of flaps was based on the size and the preoperative dimensions of the defect.

### Flap Design

**Fig. 1:**
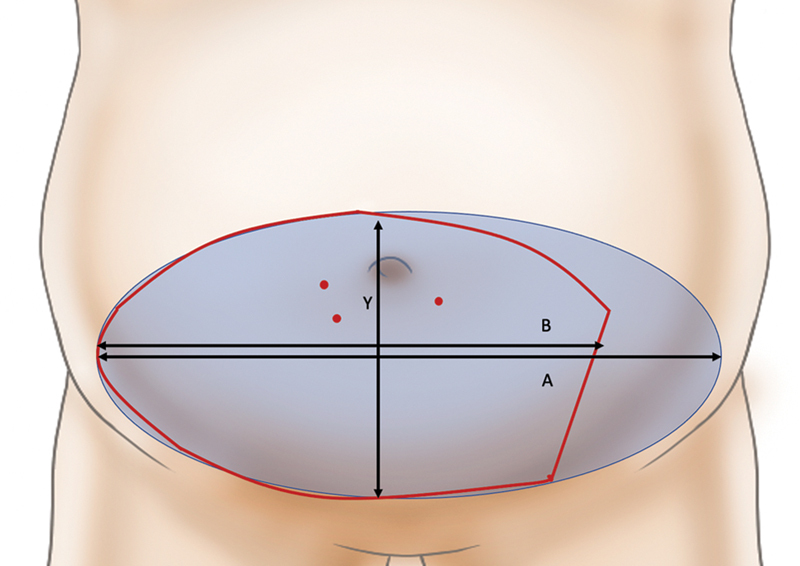
The flap is designed transversely around the largest perforator and is the same size as the defect (B). An additional transverse full ellipse (A). If the ratio B/A was greater than 0.75, a bipedicle deep inferior epigastric perforator (DIEP) flap is considered.

The flap was designed transversely around the largest perforator and was the same size as the defect (B). An additional transverse full ellipse (A) was drawn with the same vertical height of the defect along the transverse axis. For primary closure of the donor site, the lower incision line was set slightly higher than that of a breast DIEP flap. We confirmed that closure of the donor site is possible in cases at standing position before the surgery through pinching. We calculated the ratio of these dimensions (B/A); if the ratio was greater than 0.75, we decided to harvest a bipedicle DIEP flap ( Fig. 1 ).

### Operative Procedure

**Fig. 2:**
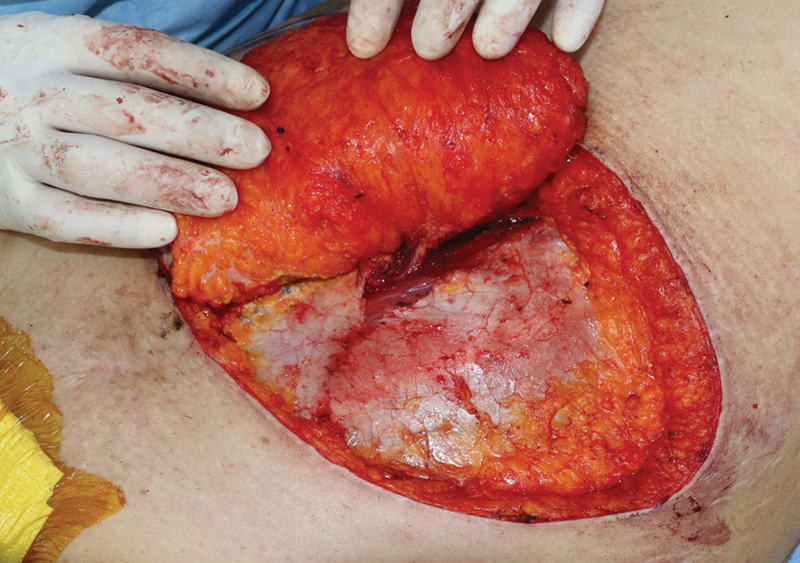
Flap elevation is performed laterally to medially on the surface of the fascia. If perforators with a sufficient caliber are chosen, the anterior rectus sheath can be incised several millimeters around the perforators.

The operative procedure is the same as DIEP in BR. The flap is elevated laterally to medially on the surface of the fascia. When elevation reaches around the target perforators, care must be taken to not damage them. If perforators with a sufficient caliber are chosen, the anterior rectus sheath can be incised several millimeters around the perforators. Then, the sheath is incised longitudinally without damaging the rectus muscle ( Fig. 2 ). The muscle is longitudinally separated according to the direction of the fibers. Meticulous perforator dissection is performed until it has been confirmed that the perforator has joined a branch of the deep inferior epigastric vessels. The motor nerves supplying the rectus abdominis muscle should always be spared. The pedicle is dissected in accordance with its required length, with efforts to preserve the nerves. Primary closure of the donor site is performed in all cases. Then, the vessels are anastomosed and the inserted flap is trimmed to fit the defect after the flap thinning procedure. If intraflap anastomosis of the bipedicle is required, it is performed after main vessel anastomosis. In two cases, the perfusion was evaluated with indocyanine green angiography.

Making a thin flap is an essential procedure to cover the large defect with DIEP flap. Various methods have been used to obtain thin flaps. A recent technique involves the harvest of a thin flap with a modified elevation plane. 1 It elevates the flap along the suprascarpal plane between the deep and superficial fat layer. However, it is technically difficult to visualize and dissect the perforators using this method. Therefore, it should be used by experienced microsurgeons.

The other method is flap thinning after conventional elevation that can be used with success if performed correctly. 5 To begin with, a perforator flap is harvested above the fascia in a traditional manner. On completion of elevation or anastomosis, the deep fat layer is removed with clear identification and protection of perforating vessels. This technique is useful if customized thinning of the flap is desired.

We performed “defatting after elevation” to remove the deep fat layer and part of the superficial fat layer was customized as per the defect. After secure microvascular anastomosis, the flap was inset temporarily and the thinning procedure was performed. Primary flap defatting was performed with scissors, in what is known as the lobule-by-lobule technique, depending on the thickness of the defect. 6 It was performed while giving due consideration to the circulation of the flap, maintenance of perfusion, and removal of loose areolar deep fat and some superficial fat under a loupe magnification. The flap thinning procedure for DIEP flaps should not only reduce thickness, but also change the shape and pliability to provide better aesthetic and functional outcomes.

## Results

Six patients (3 women and 3 men) with tissue defects in their extremities were treated with DIEP flaps and followed up for more than 36 months (mean follow-up: 61.2 months). The average age was 52 years (range: 42–64 years). The causes of the defect were trauma in five cases and diabetic foot in one case. The dimensions of the defects ranged from 180 (15 cm × 12 cm) to 480 cm ^2^ (30 cm × 16 cm) (mean: 334.6 cm ^2^ ). Intraflap crossover anastomosis was performed in four cases according to the inset rate (range: 64–100%). One artery and two venous anastomoses were performed in all cases ( Table 1 ). Primary donor site closure was accomplished in all of the patients. The postoperative course was uneventful in most of the cases. All flaps survived, except in one case of partial necrosis, which occurred in the patient with a long vertical cesarean section scar. This flap showed mild congestion at the early postoperative period, which led to partial loss that healed by secondary intention. All reconstructed extremities had satisfactory functional and aesthetic outcomes, and the donor sites healed well in all cases without complications.

**Table 1:** Patients' characteristics and outcomes

Case	Age/sex	Defect location	Comorbidities	Etiology	Recipient	Size of flap	Perforator number	Anastomosis	Bipedicled or not	Inset rate	Complications	Follow-up (mo)
1	M/ 48	Plantar and heel	Diabetes	Diabetic foot	Anterior tibial artery	320 cm ^2^ (32 cm × 10 cm)	2	1 artery (end-to-side anastomosis) 2 veins	No	70%	Wound dehiscence	65
2	F/47	Thigh and knee	None	Car accident	Inferior medial genicular artery	480 cm ^2^ (30 cm × 16 cm)	3	1 artery (end-to-side) 2 veins	Yes	80%	Partial necrosis	42
3	M/53	Plantar and heel	None	Fall-down	Posterior tibial artery	180 cm ^2^ (15 cm × 12 cm)	2	1 artery (end-to-end anastomosis) 2 veins	No	64%	No (combined both pelvic bone, femur, and tibia fracture)	66
4	F/42	Plantar and heel	None	Car accident	Posterior tibial artery	390 cm ^2^ (30 cm × 13 cm)	2	1 artery (end-to-end anastomosis) 2 veins	Yes	100%	No	65
5	M/64	Shoulder, right	Hypertension	Fall- down	Thoracoacromial artery	247.5 cm ^2^ (22.5 cm × 11cm)	3	1 artery (end-to-end anastomosis) 2 veins	Yes	92%	No (combined scapula fracture, brachial plexus)	66
6	M/58	Total hand degloving	None	Roller	Radial artery	390 cm ^2^ (30 cm × 13 cm)	3	1 artery (end-to-side anastomosis) 2 veins	Yes	100%	No	63

Abbreviations: F, female; M, male.

### Cases

#### Case 1

**Fig. 3:**
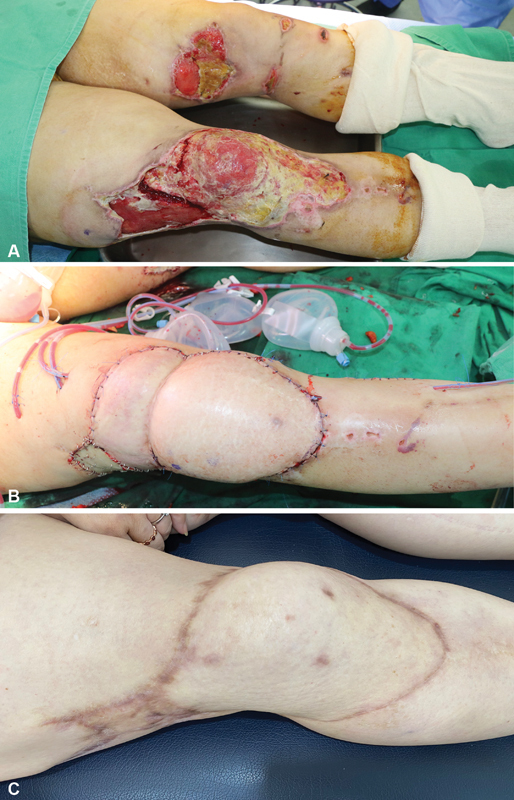
( **A** ) A skin and soft tissue defect in right upper thigh and knee area. ( **B** ) Coverage with a deep inferior epigastric perforator (DIEP) flap. ( **C** ) Long-term follow-up with full range of motion (ROM).

A 47-year-old woman suffered from a skin defect of the entire upper thigh to knee area of the right lower limb caused by a road traffic accident. She was referred to our department after internal fixation of a compound femur fracture. After debridement, the skin defect measured approximately 30 cm × 16 cm. A free DIEP flap with three perforators (two ipsilateral medial row perforators and one contralateral medial row perforator) was designed based on preoperative CTA. The patient had a long vertical abdominal scar from a cesarean section performed when she was 29 years old. The flap was elevated with three perforators of both medial rows and anastomosed in a bipedicled pattern. About 80% of the full dimension (B/A) was insetted and a thinning procedure was performed. The postoperative course was uneventful; she had a full range of motion of the knee ( Fig. 3 ).

#### Case 2

**Fig. 4:**
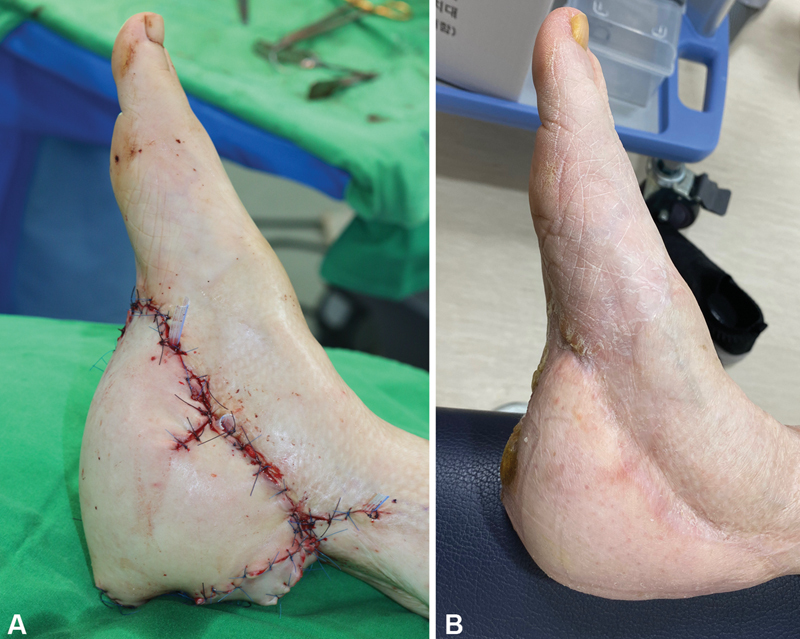
( **A** ) Coverage with a deep inferior epigastric perforator (DIEP) flap on a weight-bearing area. ( **B** ) Postoperative 25-month follow-up.

A 42-year-old woman had a car accident on the way to work and had a complicated fracture of her left foot. She visited our hospital 15 years ago for open reduction and internal fixation to fix multiple fracture of foot. A latissimus dorsi free flap was utilized to cover a skin and soft tissue defect on the plantar area. However, the flap did not survive. Finally, the weight-bearing heel was covered with a split-thickness skin graft where the calcaneal bone was exposed. Repetitive open wounds occurred with walking and contracture of the posterior calf area. After 10 years of failure of the free flap, we planned to cover the whole defect with another free flap. The dimensions that were decided were 30 cm × 13 cm. We planned to use a DIEP free flap with a bipedicled pattern because approximately100% of the full dimension (B/A) of the flap was to be insetted. The flap was elevated with two perforators of both medial rows and intraflap anastomosis was performed. The vessel was anastomosed with the posterior tibial vessels. The flap was insetted 100% after the flap thinning procedure. The postoperative course was uneventful except for temporary flap congestion. Two weeks later, the patient was discharged from the hospital. She was able to walk while wearing normal shoes ( Fig. 4 ).

## Discussion

Adequate soft tissue reconstruction in large defects of extremities is particularly important to avoid unnecessary amputation and to aid in recovery of function. The advent of perforator flaps has allowed for a more “like-for-like” replacement of full-thickness skin defects with the same tissue type, even with large defects. They permit low morbidity of the donor site, versatility in flap design, and muscle preservation with less functional deficits; additionally, the texture is similar to that of the recipient site, which provides good aesthetic results. 7 8 Selection of a perforator flap is determined by factors including reliable vasculature, acceptable donor site, reproducibility, and efficiency of procedure and complication rates. 9 10 11 12 13 In this study, we applied the classic DIEP flap, which has been frequently used for BR without design verification, similar to that in other studies. We recommend it for large defects after some prior surgical requirements: preoperative planning for perforator selection to avoid unexpected complications; preoperative design to support zone IV and lateral extension of the flap; preoperative confirmation of donor site closure in the standing position; intraoperative meticulous dissection of perforators; additional vascular support with additional anastomosis; and individualized defatting for the defect.

The DIEP flap has become the first choice for autologous BR since it was introduced by Koshima et al in 1992. 14 It provides a large amount of tissue, relatively easy dissection during flap elevation, and minimal donor site morbidity with concealed scar at lower abdomen. 13 15 16 17 18 Despite these advantages, it has rarely been used in areas other than the breast according to previous literature; it has been rarely reported for extremity reconstruction since the 2000s. A case study introduced a reconstruction with a DIEP flap in diabetic foot ulceration in 2005. 19 In the same year, Masuoka et al introduced the method for foot reconstruction, successfully using an exterior pedicle in two cases. 20 Landuyt et al presented 25 cases of DIEP flaps in lower extremity reconstruction including a pedicled flap. They conducted the largest study using DIEP flaps, proving that apart from its merits in BR, it offers diverse opportunities. In the 2010s, several case series reported good results of the DIEP flap for extremity reconstruction in children. 21 22 23 A study that used the chimeric DIEP flap for the reconstruction of posttraumatic drop foot deformity, inter alia, reported that two patients recovered active dorsiflexion of the ankle after the surgery. 23 Two studies that compared DIEP and other free flaps (anterolateral thigh perforator flap) 24 and circumflex scapular artery perforator flap 25 for the reconstruction of lower extremities in pediatric patients concluded that the DIEP flap might be a good alternative for foot reconstruction; however, other flaps showed better morpho-functional outcomes than the DIEP flap. They embraced it as a second option for selected cases in extremity reconstruction and do not explain the technical details.

DIEP might not be primarily selected to reconstruct large defects of the extremities and we considered several technical details to overcome the weaknesses of the DIEP flap, including the relatively high rate of perfusion-related problems which result in flap necrosis, and its bulkiness, which required “thickness-controlling.”

**Fig. 5:**
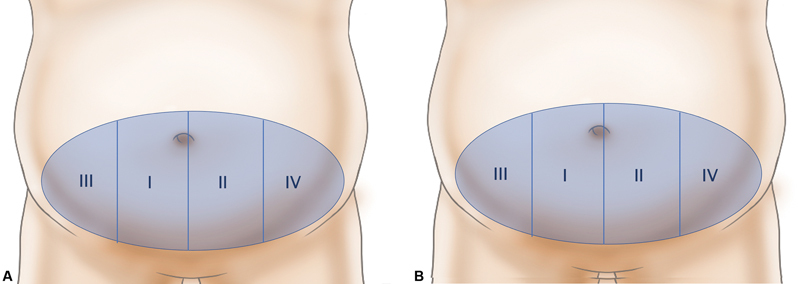
( **A** ) Hartlampf perfusion zone. ( **B** ) Holm perfusion zone.

Unless you are a breast surgeon, vascular anatomy might be an unfamiliar territory, which may lead to perfusion-related problems and disadvantages due to bulkiness. When DIEP flaps are used, discarding tissue with unreliable perfusion is crucial to prevent perfusion-related complications. Classically, the so-called Hartlampf perfusion zone is commonly used to decide the discarded zone ( Fig. 5 ). Holm et al recommended new perfusion zones based on their clinical fluorescent studies and suggested that zones II and III be reversed ( Fig. 5 ). Recently, Saint-Cyr et al introduced a perfusion zone concept that varies depending on location, rows, and quality of the perforators. 26 They showed that medial and lateral row perforators have different perfusion patterns. The classical Hartlampf concept for the medial perforator trace and the Holm's concept for the lateral row perforators have been recommended. It is the latest model for perfusion pattern analysis of the DIEP flap after minor modifications 27 28 that should be considered to perform safe DIEP flap coverage.

After understanding of these concepts, the circulatory problems of DIEP flap can be sufficiently overcome with an additional surgical procedure. Providing additional vascular flow between pedicles enables us to augment the territory of the flap. As a result, an extra-arterial inflow (bilateral pedicles or combination with superficial epigastric vessels) can be charged to incorporate more lateral skin without any circulatory problems. Understanding of the circulatory zone of the DIEP flap and the design of flap should be individualized according to the location of the perforators (medial or lateral). These procedures could allow for an extended skin flap by supporting zone IV, which can be planned before the surgery.

Another major reason the DIEP flap is not widely chosen is its “bulkiness.” If an appropriate defatting procedure is not achieved, a flap larger than the defect might be needed and aesthetic insetting will not be possible. Defatting, or thinning procedure, means the removal of tissue in the deep subcutaneous fat layer, superficial fascia system, and part of the superficial subcutaneous fat layer, except the area around the perforators. 29 The superficial fascial system supports the fat and holds the skin onto the underlying tissues. 30 Thus, disruption of the superficial fascial system could change the integrity of the superficial fat layer and the flap becomes more pliable and stretchable. We performed conventional perforator flap elevation at the suprafascial plane and flap defatting was performed with scissors in what is known as the lobule-by-lobule technique after microanastomosis, according to the required thickness. 31 This procedure allows a flap to be insetted favorably to curved defects in the extremities without needing to design a larger flap. Controlling flap thickness is an essential procedure to overcome the weaknesses of the DIEP flap and to provide successful coverage in large defects. Furthermore, it results in high patient satisfaction with substantial aesthetic improvement. 29

Although this study includes successful functional and aesthetic results with DIEP flaps for large extremity defects through long-term follow-up, the limitations of a retrospective study and a small number of patients exists.

Advancements in microsurgical techniques and various perforator flaps have provided better functional and aesthetic outcomes for large defects in extremities. The DIEP flap is not the first choice for soft tissue defect coverage for large defects; however, it could be an alternative option for reconstruction with “one-stage perforator flap” based on the largest dimension, low donor site morbidity, and concealed scar.
